# Physiotherapy for Urinary Incontinence After Radical Prostatectomy: Current Evidence and Therapeutic Challenges

**DOI:** 10.3390/jcm15187245

**Published:** 2026-09-17

**Authors:** Izabela Mastej-Stawarska, Edyta Łuszczki, Maciej Tomasz Kochman

**Affiliations:** Faculty of Health Sciences and Psychology, University of Rzeszów, Al. Tadeusza Rejtana 16C, 35-959 Rzeszów, Poland

**Keywords:** urinary incontinence, radical prostatectomy, physiotherapy

## Abstract

Urinary incontinence remains one of the most frequent and distressing complications after radical prostatectomy and can substantially affect quality of life. Physiotherapy, particularly pelvic floor muscle training (PFMT), is widely used as part of conservative postoperative management; however, substantial variation exists in patient populations, surgical and perioperative factors, intervention protocols, timing of initiation, adjunctive modalities, outcome definitions, and follow-up. This narrative review summarizes current evidence on pelvic floor rehabilitation after radical prostatectomy, focusing on prehabilitation, PFMT, biofeedback, electrical stimulation, neuromodulation, and emerging modalities such as transfer energy capacitive and resistive (TECAR) therapy. PFMT is the most widely studied conservative physiotherapy intervention and may accelerate early recovery of continence in some patients, although its effects on longer-term continence rates are less certain. The role of biofeedback and electrical stimulation remains unclear because of heterogeneous protocols and inconsistent outcome reporting. Evidence for neuromodulation is limited and should be interpreted according to the specific intervention and study population. TECAR therapy currently lacks direct clinical evidence supporting its efficacy in post-prostatectomy urinary incontinence. Across the literature, differences in continence definitions, intervention content and dose, baseline symptom severity, surgical techniques, timing of treatment, and follow-up complicate direct comparison and may confound observed improvements because spontaneous recovery is common after surgery. Current evidence supports PFMT as a reasonable core component of conservative management after radical prostatectomy, particularly for accelerating early continence recovery, whereas long-term superiority and the added value of adjunctive modalities remain uncertain.

## 1. Introduction

Prostate cancer is the most commonly diagnosed malignant tumour among men and is one of the leading causes of cancer-related deaths in developed countries [1]. It is the most common cancer in men in more than half of the world’s countries and the leading cause of cancer-related deaths in men in 46 countries [2]. Prostate cancer is a significant public health problem. This condition not only impedes healthy ageing but is also associated with substantial social and economic costs [3].

Although not all risk factors for prostate cancer are known, non-modifiable factors such as age, genetic predisposition and ethnic origin have been identified. Growing evidence from epidemiological and observational studies indicates that modifiable lifestyle factors, particularly diet, obesity, tobacco use, chronic infectious–inflammatory prostatic processes and occupational exposure to cadmium, herbicides and pesticides, play an important role in the development and progression of diseases, including prostate cancer [4].

Due to the location of prostate cancer growth, the initial phase of the disease is often asymptomatic. By the time urinary symptoms appear, the disease may already be in an advanced stage. Therefore, screening tests are particularly important, as they enable earlier detection of prostate cancer [5]. It should be noted that the increase in diagnosed cases of prostate cancer is a result of the introduction of the serum prostate-specific antigen (PSA) test. The glycoprotein PSA is produced by the glandular part of the prostate and is widely regarded as a marker of prostate cancer. However, elevated PSA levels are not specific to prostate cancer [5,6].

One of the primary methods of treating prostate cancer is surgical treatment in the form of radical prostatectomy (RP) and robot-assisted radical prostatectomy has become increasingly common. This involves the complete removal of the prostate and is often performed in conjunction with lymphadenectomy [7]. Despite its high efficacy in the oncological treatment of patients, radical prostatectomy may be associated with postoperative complications that affect patients’ functionality and quality of life. In most cases, postoperative sequelae take the form of urinary incontinence, pain and erectile dysfunction [8,9]. The mechanisms leading to the occurrence of these complications are multifactorial and include, among others, damage to the urethral sphincter apparatus, neurovascular bundles, as well as biomechanical disturbances within the pelvic floor [10].

The role of urological physiotherapy in the prevention, treatment and rehabilitation of urological problems is increasingly recognised [11]. The aim of the physiotherapist’s work in the field of urology is to restore normal pelvic floor muscle function, improve urinary control and minimise the negative functional consequences of surgery. Restoring impaired or lost functions through surgical treatment is crucial to improving patients’ quality of life [12].

Given the rising incidence of prostate cancer and the increasing use of radical prostatectomy, a key challenge is not only to determine whether physiotherapy can support continence recovery, but also to clarify which patients benefit, which intervention components are clinically meaningful, when rehabilitation should be initiated, and how outcomes should be standardized. For this reason, a narrative review of the literature was undertaken with the aim of providing a comprehensive understanding of the role of urological physiotherapy.

## 2. Aim and Review Method

The aim of this review is to critically synthesize current evidence on physiotherapy for post-prostatectomy urinary incontinence, with particular emphasis on pelvic floor muscle training, prehabilitation, adjunctive modalities, outcome assessment, and the methodological limitations that hinder translation into clinical practice.

This review was designed as a narrative evidence synthesis rather than a systematic review or meta-analysis. This approach was selected because the available literature includes substantially heterogeneous populations, intervention protocols, treatment timings, comparators, outcome definitions, follow-up periods, and study designs. The objective was therefore to provide a clinically oriented synthesis of the available evidence while explicitly considering its directness, methodological limitations, and applicability to men following radical prostatectomy.

The final searches were performed on 30 May 2026 in PubMed and PEDro. PubMed was searched using the following string: ((radical prostatectomy OR post-prostatectomy) AND (urinary incontinence OR stress urinary incontinence) AND (pelvic floor muscle training OR physiotherapy OR rehabilitation OR biofeedback OR electrical stimulation OR neuromodulation OR electroacupuncture OR TECAR OR radiofrequency)). PEDro was searched using ‘’radical prostatectomy’’, “pelvic floor muscle training”, “biofeedback”, “electrical stimulation” and “neuromodulation”. Google Scholar was used only as a supplementary source; the first 50 results were screened and screening was stopped when no additional relevant records were identified.

Priority was given to studies involving men following radical prostatectomy and reporting continence recovery, outcomes, pelvic floor muscle function or patient-reported outcomes. Reference lists of key publications were additionally screened to identify relevant articles. The identified literature was assessed for relevance to the predefined scope of the review. Records identified through database searches and reference-list screening were initially screened by title and abstract, followed, where appropriate, by full-text assessment. Disagreements were resolved by discussion. This narrative review did not aim to provide an exhaustive systematic identification of all eligible studies and no formal risk-of-bias assessment was performed. Duplicate records identified across the databases were removed before the relevance assessment.

To address the substantial heterogeneity of the evidence base, the literature was categorized according to study design and directness of evidence. Direct evidence comprised clinical studies conducted in men undergoing or following radical prostatectomy. Indirect evidence comprised studies conducted in other populations or clinical conditions that could provide mechanistic or hypothesis-generating information but could not establish clinical efficacy in post-prostatectomy urinary incontinence. Systematic reviews and clinical guidelines were considered contextual evidence and were interpreted separately from original clinical studies.

Rather than applying a formal risk-of-bias assessment tool, each included original clinical study underwent a structured methodological appraisal addressing study design, sample size, population characteristics, baseline continence status, intervention timing and dose, comparator, outcome definition, follow-up duration, adherence, and major methodological limitations.

Because the identified studies differed substantially in population, intervention characteristics, treatment timing, comparator, outcome definitions, and follow-up, quantitative pooling was not considered appropriate. Findings were therefore synthesized narratively and organized according to intervention type, clinical stage of rehabilitation, study design, and directness of evidence.

Where evidence was derived from populations other than men following radical prostatectomy, it was explicitly identified as indirect and was not used as direct evidence of clinical effectiveness in the post-prostatectomy population. The strength of clinical conclusions was calibrated according to the directness, consistency, and methodological limitations of the available evidence.

Because the available literature comprises randomized trials, observational studies and indirect evidence from non-post-prostatectomy populations, the evidence is summarized below according to study design and directness. Table 1A presents original clinical studies conducted in men undergoing or following radical prostatectomy, whereas Table 1B separates indirect radiofrequency/TECAR-related evidence from non-target populations. Systematic reviews and guidelines are discussed narratively and are not presented as equivalent to original clinical studies.

## 3. Post-Prostatectomy Complications

The technique of surgical treatment for prostate cancer has changed significantly over the years. In recent years, robot-assisted radical prostatectomy has become increasingly common, reflecting the growing use of minimally invasive surgical approaches in the management of localized prostate cancer [24]. Despite its numerous advantages, radical prostatectomy carries a risk of complications. The patients following radical prostatectomy most commonly experience SUI and erectile dysfunction [25]. The etiology of urinary incontinence following radical prostatectomy is not yet fully understood, but it is associated with reduced pelvic floor integrity and coordinated function of the remaining continence mechanisms, including the striated urethral sphincter, pelvic floor muscles, connective tissues, and supporting vascular structures. Radical prostatectomy removes the proximal urethra together with a substantial portion of the urethral smooth muscle and disrupts the surrounding connective tissues, thereby impairing the normal continence mechanism. Consequently, maintenance of continence relies predominantly on the residual striated urethral sphincter and pelvic floor muscles [15,26]. Damage to pelvic floor structures during surgery can lead to a predominant form of stress urinary incontinence, which manifests as involuntary urine leakage during coughing, sneezing, laughing or physical activity [16,27]. Erectile dysfunction in patients following surgical treatment is caused by the removal or damage of neurovascular bundles, which consequently manifests as haemodynamic disturbances [17,28].

Studies show that there are factors influencing urinary incontinence after surgery which are patient-related, i.e., age and body mass index (BMI), biological factors, including anatomical conditions (urethral length, condition of supporting tissues, shape and size of the prostate), and surgical factors (preservation of the bladder neck, reconstructive techniques). Furthermore, risk factors for urinary incontinence include the level of physical activity and the baseline condition of the pelvic floor muscles [18,29].

## 4. Clinical Assessment and Outcome Measures

Accurate assessment of post-prostatectomy urinary incontinence (PPI) is essential not only for documenting symptom severity and monitoring treatment response, but also for determining whether a patient is an appropriate candidate for conservative physiotherapy as the primary management strategy. A clinically useful assessment should therefore proceed from symptom classification and severity assessment to identification of patients who may require additional urological investigation or an alternative treatment pathway [19,20,21,30,31,32].

The initial evaluation should include a detailed history aimed at determining the predominant type of urinary incontinence, its severity, timing in relation to radical prostatectomy, and its impact on daily functioning and quality of life. In accordance with a decision-oriented approach, patients should be assessed with regard to the type of incontinence, severity of leakage, and degree of bother before treatment is selected. Symptoms should therefore be classified as predominantly stress urinary incontinence, mixed urinary incontinence, or urgency-predominant urinary symptoms rather than being considered a single, homogeneous form of post-prostatectomy incontinence [20,21,31,32].

Importantly, outcome measures used in clinical studies are not interchangeable, and the definition of continence and the timing of outcome assessment should be considered when interpreting treatment effects.

The diagnostic process begins with a detailed medical history, including the onset and severity of urinary leakage, frequency of episodes, precipitating activities, use of pads, previous treatment, current medication, comorbidities, and other relevant clinical factors. Symptoms should be characterised according to their predominant presentation, including stress, urgency, or mixed urinary incontinence [30,31,32].

Physical examination should complement the history and include assessment of the pelvic region, pelvic floor muscle function, and, when clinically appropriate, digital rectal and basic neurological examination. A cough stress test may help demonstrate stress-related leakage and support clinical classification of the predominant symptom pattern [30,31,32]. Assessment of pelvic floor muscle function should include the patient’s ability to identify, voluntarily contract, sustain, relax, and rapidly activate the pelvic floor muscles. Digital examination and, where available, ultrasound or other instrumental methods may assist in evaluating contraction quality; however, assessment of muscle function should be distinguished from direct measurement of urinary leakage [30,32,33]. Further urological investigation, including cystoscopy or urodynamic testing, may be appropriate when the clinical presentation raises concern regarding an alternative diagnosis, obstruction, storage dysfunction, impaired bladder emptying, or when symptoms are not adequately explained by uncomplicated stress urinary incontinence. Particular attention should be paid to clinical findings that may require urological reassessment rather than continuation of physiotherapy as the sole management strategy. These may include symptoms suggestive of anastomotic stenosis or other bladder outlet obstruction, urinary tract infection, significant urinary retention, severe or predominant urgency, unexplained or complex lower urinary tract symptoms, and persistent high-volume leakage. In such circumstances, physiotherapy should be integrated into a broader diagnostic and management pathway rather than assumed to be sufficient as a stand-alone intervention.

Objective and patient-reported measures remain important after clinical classification has been established. Pad use and pad-weight tests can quantify urinary leakage, while voiding diaries can characterise frequency, urgency, fluid intake, voided volumes, and leakage episodes. Validated patient-reported outcome measures provide complementary information regarding symptom burden and quality of life [30,34,35]. Moreover, validated questionnaires such as ICIQ-UI SF (International Consultation on Incontinence Questionnaire on Urinary Incontinence Short Form) and the ICIQ-MLUTS LF (International Consultation on Incontinence Questionnaire on Male Lower Urinary Tract Symptoms Long Form)are frequently used to assess patient-reported symptoms and their impact on quality of life [35].Quality-of-life measures and patient-reported perception of improvement may be particularly relevant when evaluating interventions because a reduction in symptoms or improvement in quality of life does not necessarily correspond to complete cessation of urinary leakage.

These outcomes should be interpreted according to the patient’s predominant symptom pattern and should not substitute for clinical assessment when the presentation suggests a condition requiring further urological evaluation.

Accordingly, assessment may be conceptualised as a stepwise decision framework. The first step is to determine the predominant symptom type, including whether the presentation is predominantly stress-related, mixed, or urgency-predominant. The second step is to assess severity and the degree of bother using appropriate objective and patient-reported measures. The third step is to identify clinical features suggesting complications, obstruction, infection, retention, or other conditions requiring urological reassessment. Only after this clinical categorisation should the most appropriate conservative rehabilitation strategy be selected.

This approach is particularly relevant when interpreting physiotherapy studies. Trials that include patients with different baseline symptom patterns, severity, and underlying mechanisms should not automatically be considered clinically comparable. Clear reporting of baseline continence type and severity is therefore necessary both for appropriate patient selection and for interpretation of treatment outcomes.

Despite the availability of numerous assessment tools, considerable heterogeneity exists across clinical studies regarding continence definitions, pad-test protocols, follow-up duration, and the choice of patient-reported outcome measures. Greater standardisation of outcome assessment would improve comparability between studies and strengthen the evidence supporting physiotherapy interventions after radical prostatectomy.

A major methodological issue in the evidence synthesis is the lack of a uniform definition of continence. Across studies, continence may be defined as complete absence of pad use, use of no pads or only one safety pad per day, a predefined threshold of urinary loss on a pad test, achievement of a specific questionnaire score, or patient-reported recovery of urinary control. These definitions represent different outcomes and may produce different estimates of treatment effectiveness. For example, a patient who uses one safety pad per day may be classified as continent in one study and incontinent in another. Similarly, improvement in an ICIQ-UI SF score represents a change in patient-reported symptom burden and should not be considered equivalent to objective absence of urine leakage.

The timing of outcome assessment is equally important. Early outcomes, such as time to continence or the proportion of patients achieving continence within the first weeks or months after catheter removal, reflect the speed of recovery. These outcomes should therefore be analysed separately and should not be combined into a single measure of treatment efficacy. A treatment may shorten the time to continence without producing a clinically important difference in the final proportion of continent patients at longer-term follow-up.

Objective leakage outcomes, including pad weight or clearly defined pad-use criteria, should also be distinguished from patient-reported symptom and quality-of-life outcomes. Neither type of outcome should automatically be considered a substitute for the other.

For this reason, greater standardisation of outcome definitions and reporting is needed in future studies of physiotherapy after radical prostatectomy. At a minimum, studies should clearly define continence before analysis, specify the measurement method and follow-up time, report both absolute and relative treatment effects where appropriate, and distinguish objective urinary leakage from patient-reported symptoms and quality-of-life outcomes. Such standardisation would improve comparability between trials and reduce the risk of drawing misleading conclusions when synthesising heterogeneous evidence.

## 5. Physiotherapy Interventions

Physiotherapy is widely incorporated into the multidisciplinary management of patients with genitourinary disorders [36]. Following radical prostatectomy, pelvic floor muscle training (PFMT) aims to support the restoration of pelvic floor muscle function and improve the patient’s ability to activate the muscles involved in urinary continence [36,37]. Surgical disruption of the prostate and surrounding anatomical structures may alter the function and coordination of the continence mechanism. PFMT is therefore intended to improve pelvic floor muscle strength, endurance, coordination, and the ability to generate a timely contraction during increases in intra-abdominal pressure [38,39]. However, the precise mechanisms through which PFMT contributes to continence recovery remain incompletely understood, and individual responses to rehabilitation may vary considerably.

A further limitation is the lack of standardisation of physiotherapy and prehabilitation programmes. Studies differ substantially with regard to the timing of intervention, exercise frequency, session duration, progression criteria, supervision, and use of adjunctive techniques. This heterogeneity limits direct comparison between studies and makes it difficult to define a single optimal rehabilitation protocol or formulate universally applicable recommendations.

### 5.1. Pelvic Floor Muscle Training

Pelvic floor muscle training (PFMT) is the most widely studied conservative physiotherapy intervention following radical prostatectomy and remains an important component of postoperative continence rehabilitation [40]. Its rationale is based on improving the strength, endurance, coordination, and timely activation of the pelvic floor musculature, which may contribute to compensatory support of the continence mechanism following surgery [40,41]. PFMT may also be combined with adjunctive interventions, including electromyographic (EMG) biofeedback [41] or electrical stimulation (ES) [42].

Nevertheless, despite the extensive literature on PFMT, the evidence should be interpreted with caution. The Cochrane review (2023) concluded that the overall value of conservative interventions for post-prostatectomy urinary incontinence remains uncertain, and the effects of PFMT combined with biofeedback were also considered uncertain. The review additionally identified insufficient evidence to establish the effects of electrical stimulation for several clinically relevant outcomes [38]. Thus, although PFMT is widely used and has a strong physiological rationale, the available evidence does not justify describing it as unequivocally superior to all other conservative interventions.

For PFMT, an important initial step is verification that the patient can correctly identify and voluntarily contract the pelvic floor muscles. Studies should therefore specify whether correct contraction was assessed using digital rectal examination, ultrasound imaging, electromyographic guidance, or another method, and whether compensatory activation of the abdominal, gluteal, or hip muscles was assessed [13,40,41,42].

The lack of standardisation further limits interpretation of the available evidence. Differences in the frequency of sets (from 1 to 3 per day), session duration (25–60 min) and the method of exercise progression (number of repetitions and sets, contraction time) significantly hinder the interpretation of results and their implementation in clinical practice, which may result in insufficient effectiveness of the therapies provided [13,29,30,31,32,33,40,41,42,43].

The role of therapist supervision is similarly not fully established. Some studies suggest that supervised PFMT may improve early continence outcomes compared with unsupervised programmes, potentially because professional instruction can facilitate correct identification and selective activation of the pelvic floor muscles [43]. However, the evidence remains heterogeneous, and the EAU guidance describes the role of supervised PFMT as controversial [44]. Therefore, supervised PFMT may be considered a potentially beneficial approach for selected patients rather than being regarded as consistently superior to unsupervised exercise.

PFMT should therefore be viewed not as a uniform intervention but as an individualized rehabilitation programme adapted to the patient’s baseline pelvic floor function, ability to perform the exercises correctly, motivation, adherence, and postoperative recovery.

Overall, PFMT remains a reasonable and commonly recommended component of conservative management after radical prostatectomy, particularly during the early postoperative period. Importantly, recommending or offering PFMT as part of conservative postoperative management should not be interpreted as evidence that any specific PFMT protocol, mode of delivery, or adjunctive intervention has been shown to be unequivocally superior to alternatives. Its potential benefit appears most consistently related to the timing of continence recovery, whereas evidence for a sustained improvement in continence rates at later follow-up is less certain. Future randomized controlled trials should use standardized intervention protocols, clearly defined exercise parameters, objective measures of adherence, and consistent outcome definitions, while reporting both time-to-continence and continence rates at predefined longer-term follow-up points.

### 5.2. Timing of Intervention and Prehabilitation

The optimal timing of pelvic floor rehabilitation after radical prostatectomy has not been definitively established [45]. The timing of intervention should therefore be described according to clinically distinct phases rather than by assigning a single postoperative time point to all patients.

Prehabilitation refers to PFMT initiated before radical prostatectomy. Evidence regarding preoperative PFMT is heterogeneous, although some studies suggest that prehabilitation may improve early postoperative continence recovery in selected patients [14,15,35,36,37,45]. These findings should nevertheless be interpreted cautiously because studies differ in intervention duration, training intensity, patient characteristics, and outcome definitions. Prehabilitation should therefore be regarded as a potentially beneficial strategy rather than as an intervention with a uniformly established effect on long-term continence.

Postoperative PFMT is generally introduced after removal of the urinary catheter or according to the patient’s postoperative clinical status and the treating team’s protocol [15]. Early postoperative rehabilitation may be associated with faster recovery of continence in some studies; however, the available evidence does not establish a single universally optimal starting point. Accordingly, postoperative PFMT should be initiated with consideration of surgical recovery, catheter removal, the patient’s ability to perform the exercises correctly, and individual clinical circumstances rather than according to an arbitrary postoperative time threshold.

For patients with persistent urinary incontinence, rehabilitation should be reassessed rather than continued indefinitely without evaluation of the underlying cause and severity of symptoms. Persistent or severe incontinence despite an appropriate period of conservative management may warrant further urological assessment and discussion of additional treatment options, including surgical management when clinically indicated. The timing of such escalation should be based on symptom severity, degree of bother, recovery trajectory, and current guideline recommendations rather than on a single fixed time point.

Taken together, the available evidence supports distinguishing four clinical stages: prehabilitation before surgery, early postoperative rehabilitation following catheter removal, continued rehabilitation during the period of spontaneous recovery, and reassessment of persistent incontinence when recovery is incomplete. This framework avoids assigning unsupported fixed time windows to physiotherapy while allowing treatment timing to be adapted to the individual patient’s clinical course.

### 5.3. Biofeedback

EMG biofeedback is used as an adjunct to PFMT to facilitate identification and activation of the pelvic floor muscles [16]. Biofeedback may facilitate correct pelvic floor muscle activation and may be useful in selected patients, particularly when voluntary contraction is difficult to identify. However, its incremental benefit over well-instructed PFMT remains uncertain [16,17].

Direct evidence in men after radical prostatectomy remains limited and heterogeneous, and available studies differ in device type, electrode/probe placement, treatment purpose and assessment reliability. Furthermore, the lack of specific guidelines on how to conduct EMG biofeedback further complicates the interpretation of the results obtained. Accordingly, biofeedback should currently be considered an adjunct to PFMT rather than a replacement for PFMT.

### 5.4. Electrical Stimulation

Electrical stimulation is a non-invasive form of therapy that has become increasingly important in recent years for the treatment of patients with lower urinary tract dysfunction. Electrical stimulation (ES) of the pelvic floor muscles involves stimulating the pudendal nerve and its branches, leading to the direct activation of the striated muscles of the urethra and periurethral region. It may also elicit reflex muscle responses within the pelvic floor [18,19]. ES is sometimes combined with PFMT or EMG biofeedback; however, whether these combinations provide clinically meaningful additional benefit remains uncertain [46].

Despite a growing number of reports indicating the potential efficacy of electrical stimulation in reducing the symptoms of urinary incontinence, the results of studies are inconclusive. A significant limitation is the considerable heterogeneity of the interventions used, which include various forms of stimulation (transcutaneous, transrectal), the lack of specified treatment parameters and duration (ranging from 15 to 20 min), which makes it difficult to directly compare results and draw clear clinical conclusions [47].

Because the available studies differ substantially in frequency, intensity, electrode placement, treatment schedule, and duration, the term, ”electrical stimulation” encompasses a heterogeneous group of interventions. Results from different protocols should therefore not be interpreted as evidence for a single, standardised electrical stimulation treatment.

### 5.5. Neuromodulation

Neuromodulation methods, including nerve stimulation and electroacupuncture, have shown potential efficacy in selected patient groups; however, the available evidence remains limited compared with that for conventional conservative therapies.

Nerve stimulation may modulate neural pathways involved in lower urinary tract function and pelvic floor control and may therefore contribute to improved urinary continence [48]. In a retrospective cohort study of patients with post-radical prostatectomy incontinence, electrical pudendal nerve stimulation (EPNS) was associated with greater short-term improvements in continence outcomes compared with pelvic floor muscle training combined with transrectal electrical stimulation (PFMT + FES) [20]. Nevertheless, the retrospective design and limited follow-up preclude firm conclusions regarding long-term effectiveness, and further comparative studies are warranted.

Electroacupuncture should be considered separately from sacral neuromodulation. Electroacupuncture combines traditional acupuncture with electrical stimulation and targets acupuncture points corresponding to anatomical regions associated with the sacral micturition centre. Importantly, one directly relevant randomized controlled trial has specifically evaluated electroacupuncture in patients with early urinary incontinence following radical prostatectomy. The study included 110 participants and used sham stimulation as the control intervention [21]. This trial provides preliminary direct evidence that electroacupuncture may have a therapeutic role in the management of post-prostatectomy urinary incontinence; however, evidence from a single RCT is insufficient to establish its effectiveness or support broad clinical implementation. Independent replication in further well-designed trials is therefore required, particularly with longer follow-up and standardized outcome measures.

Overall, neuromodulation and electroacupuncture may represent promising adjunctive approaches in the management of post-prostatectomy urinary incontinence. However, the evidence base remains limited, and the findings for electroacupuncture currently rely on one directly relevant RCT that requires replication. Further well-designed randomized controlled trials with clearly defined clinical endpoints, adequate follow-up, standardized treatment protocols, and systematic assessment of adverse events are needed before these approaches can be considered for routine use.

### 5.6. Emerging Methods (e.g., TECAR)

Radiofrequency therapy, including TECAR (transfer energy capacitive and resistive), is an emerging physiotherapeutic intervention whose use has been proposed in the management of pelvic floor dysfunction. However, there is currently no direct clinical evidence demonstrating its efficacy for urinary incontinence in men following radical prostatectomy. The available studies provide only indirect evidence, as they have been conducted in populations that differ from the target population.

The evidence cited in the current manuscript includes a study of monopolar capacitive-resistive radiofrequency in 40 women with stress urinary incontinence [22]. This study did not include men following radical prostatectomy and therefore cannot be used as direct evidence of the effectiveness of TECAR in post-prostatectomy urinary incontinence. Similarly, the study reported in [23] investigated patients with overactive bladder and urgency urinary incontinence and was a small, single-arm pilot study. Although these studies may provide preliminary information regarding the physiological effects or feasibility of radiofrequency-based interventions, their findings cannot be extrapolated directly to men with post-prostatectomy urinary incontinence.

From a physiological perspective, radiofrequency therapy has been proposed to influence local tissue perfusion, oxygen delivery, lymphatic drainage, cellular metabolism, and connective tissue remodeling [22,23]. These mechanisms may be biologically relevant to postoperative rehabilitation, particularly in relation to tissue remodeling and local physiological changes following surgery. However, such proposed mechanisms constitute mechanistic hypotheses rather than evidence of clinical effectiveness. Given the differences between the study populations and men undergoing radical prostatectomy, it is not possible to determine whether these physiological effects translate into improved continence outcomes in the target population.

Accordingly, TECAR should currently be regarded as an experimental, hypothesis-generating intervention in the context of post-prostatectomy urinary incontinence. There is insufficient direct evidence to conclude that TECAR improves continence, accelerates recovery, or provides any other clinically meaningful benefit in men following radical prostatectomy. The available indirect studies should therefore not be interpreted as evidence supporting its clinical efficacy in this population.

Before TECAR can be considered for routine clinical use in men following radical prostatectomy, adequately designed clinical studies in the target population are required. Future research should evaluate clinically meaningful continence outcomes and establish appropriate treatment parameters, treatment frequency and duration, anatomical application, safety, contraindications, and potential adverse effects. Until such evidence is available, no specific therapeutic role or clinical benefit of TECAR in post-prostatectomy urinary incontinence can be established.

A summary of the above interventions is presented below in Table 2.

## 6. Clinical Implications and Research Gaps

The evidence base is limited by small sample sizes, short follow-up periods and substantial heterogeneity in terms of the interventions and protocols used, as well as the quality of available scientific evidence. The lack of standardisation of therapeutic protocols further hinders the synthesis of results and the development of consistent clinical guidelines and standards of physiotherapy practice following radical prostatectomy.

Several important research gaps remain. Future studies should establish standardized rehabilitation protocols with clearly defined exercise parameters, including timing of initiation, training intensity, frequency, progression, and duration of therapy. Greater consistency is also required in outcome assessment, particularly regarding definitions of continence, pad-test methodology, and patient-reported outcome measures. In addition, adequately powered randomized controlled trials with longer follow-up periods are needed to determine the durability of treatment effects and to identify patient subgroups most likely to benefit from specific rehabilitation strategies. In particular, direct clinical studies evaluating radiofrequency/TECAR therapy in men after radical prostatectomy are lacking. Addressing these methodological limitations will strengthen the evidence base and facilitate the development of more precise clinical recommendations.

## 7. Conclusions

Post-prostatectomy urinary incontinence is a clinically relevant complication for which conservative pelvic floor rehabilitation remains an important component of postoperative management. However, the available evidence is characterized by substantial heterogeneity in patient populations, surgical approaches, intervention timing and dose, treatment protocols, adherence, outcome definitions, and follow-up duration. Therefore, findings from individual studies should not be interpreted as evidence for a single standardized physiotherapy approach.

PFMT remains a reasonable core component of conservative rehabilitation and may contribute to earlier recovery of urinary continence in some patients. However, the available evidence does not establish a universally optimal timing, supervision model, exercise dose, or protocol, and evidence for a sustained improvement in long-term continence rates remains less certain. Importantly, evidence of faster early recovery should be distinguished from evidence of a higher long-term continence rate.

The incremental benefit of adjunctive biofeedback and electrical stimulation over well-instructed PFMT or behavioural therapy remains uncertain, with results varying according to the specific protocol, treatment parameters, and patient population. Neuromodulation and electroacupuncture have generated preliminary evidence of potential benefit, but the current evidence base is insufficient to support routine use and requires confirmation in adequately powered, methodologically rigorous trials with standardized outcomes and longer follow-up.

For radiofrequency-based interventions, including TECAR, no direct clinical evidence currently demonstrates efficacy for urinary incontinence in men following radical prostatectomy. Evidence from non-post-prostatectomy populations should therefore be considered hypothesis-generating rather than evidence of clinical effectiveness in the target population.

The literature is limited by heterogeneity in patient selection, intervention content, timing of initiation, session dose, outcome definitions, and follow-up duration.

Overall, the principal challenge is no longer simply to establish whether pelvic floor rehabilitation can be beneficial, but to determine which patients benefit, from which intervention, at what stage of recovery, and according to which standardized treatment protocol. Future research should prioritize adequately standardized PFMT protocols, objective adherence assessment and long-term follow-up.

## 8. Limitations

This review has several limitations. First, it was conducted as a narrative review and therefore does not provide the methodological reproducibility of a systematic review. Second, the search was limited to English-language publications indexed in selected databases. Third, no formal risk-of-bias assessment or meta-analysis was performed. In addition, the available evidence is heterogeneous with respect to patient selection, rehabilitation timing, treatment dose, intervention content, adherence and outcome assessment. These differences limit direct comparison between studies and reduce the certainty with which specific rehabilitation protocols can be recommended.

Finally, some emerging interventions discussed in the review are supported primarily by indirect or preliminary evidence. Accordingly, the conclusions should be interpreted as a critical synthesis of the available literature rather than as quantitative evidence of comparative effectiveness.

## Figures and Tables

**Table 1:** (**A**). Study design and directness from original clinical studies in men undergoing or following radical prostatectomy. (**B**) Indirect radiofrequency/TECAR-related evidence from non-post-prostatectomy populations.

**(A) Study Design and Directness from Original Clinical Studies in Men Undergoing or Following Radical Prostatectomy**						
**Study/Manuscript Reference**	**Study Design, Sample Size, and Directness of Evidence**	**Population, Surgery Type and Baseline Continence Status**	**Timing, Intervention Protocol, Duration and Adherence**	**Comparator**	**Continence Definition, Follow-Up and Principal Numerical Findings**	**Adverse Events, Barriers and Major Methodological Limitations**
García-Sánchez et al. [10]	Randomized open trial; *N* = 62; direct perioperative/preoperative evidence.	Men awaiting robotic radical prostatectomy (RARP). Baseline continence status before surgery was not clearly reported.	Guided PFMT delivered before RARP by a physiotherapist: consisting of 3 in-person sessions in which the patient was trained to perform 3 sets of 10 repetitions of slow contractions (3 s of contraction and 6 s of relaxation) with progressive (up to 5 s of contraction and 10 s of relaxation) followed by 3 fast contractions. Adherence assessment: Not explicitly reported or quantified. After surgery, both groups followed the same centre protocol for urinary incontinence management.	Written information only.	Primary outcome: continence rate measured using the pad test and ICIQ-SF. Follow-up: 1 month after robotic radical prostatectomy. No between-group difference in continence rate or incontinence severity; some quality-of-life domains favoured the written-information group.	Adverse events: NR. Limitations: open-label design, short initial follow-up, limited protocol/adherence reporting. Interpretation: does not support a strong claim that guided preoperative PFMT is superior to written instructions.
de Lira et al. [11]	Randomized clinical trial; 31 men randomized from 59 eligible: control n = 15, physical therapy n = 16; direct perioperative PFMT evidence.	Men undergoing radical prostatectomy; preoperative urinary incontinence was an exclusion criterion.	Two pre-RP physiotherapist-guided PFMT sessions including exercises and EMG biofeedback, plus verbal/written instructions to continue PFMT until surgery and resume after catheter removal. Adherence assessment: NR.	Usual post-RP care.	Outcomes: ICIQ-SF and IIEF-5 at 3 months after RP. UI rate: 72.7% vs. 70.0%. ICIQ-SF: 6.9 ± 6.26 vs. 7.0 ± 5.12 (*p* = 0.97). No significant improvement in early continence or erectile function.	Adverse events: NR. Limitations: very small sample, short follow-up, only two supervised preoperative sessions. Interpretation: mixed/negative evidence for a brief perioperative PFMT protocol.
Sehgal et al. [13]	Retrospective comparative analysis; N = 56; direct observational evidence, not an intervention RCT.	Men from a prostate cancer rehabilitation clinic; evenly split between open RP and robotic-assisted laparoscopic prostatectomy. Baseline characteristics and patient-reported UI symptoms were measured preoperatively.	Physiotherapy for UI after RP in a rapid-access rehabilitation pathway. Two sessions: initial assessment, internal examination, individualized PFMT/home programme and mirror-based biofeedback, followed by a review session with treatment modification as needed. Additional sessions were optional and self-funded. Participants had completed ≥2 sessions by 9 months post-surgery. Adherence: Inclusion required completion of at least two physiotherapy sessions by 9 months post-surgery; adherence to the home exercise programme was not reported.	Comparison by surgical approach: open vs. robotic-assisted laparoscopic RP. No untreated/non-physiotherapy control group.	Outcome: ICIQ-UI global score. Follow-up: 3 months and 2 years post-surgery. No significant differences between open and robotic subgroups at 3 months; both subgroups improved between 3 months and 2 years. Physiotherapy did not differentially affect open vs. robotic-assisted subgroups.	Adverse events: NR. Limitations: retrospective design, no untreated control, non-standardized intervention reporting, possible confounding by surgical and patient factors. Interpretation: useful for postoperative rehabilitation outcomes, not for comparative efficacy.
Centeme ro et al. [14]	Prospective randomized study; N = 118; preoperative + postoperative PFMT n = 59 vs. postoperative-only PFMT n = 59; direct prehabilitation evidence.	Men with localized prostate cancer undergoing open radical retropubic prostatectomy. Baseline continence status was not clearly reported.	Group A started PFMT 28 days before and continued postoperatively; Group B started PFMT postoperatively, with both groups receiving PFMT instruction on postoperative day 2 (catheter removal day). Adherence: All patients recorded their PFMT practice in a performance table, which was reviewed at each outpatient visit to confirm implementation status.	Postoperative PFMT only.	Primary: patient-reported continence, defined as no urinary leakage. Secondary: 24 h pad test and ICS-male SF. At 1 month: continence 44.1% vs. 20.3% (*p* = 0.018). At 3 months: 59.3% vs. 37.3% (*p* = 0.028), favouring preoperative PFMT. ICS-male SF also favoured the preoperative group.	Adverse events: NR. Limitations: single-study early benefit, open surgery only, outcome definitions and surgical pathways may differ from modern RP. Interpretation: supports possible early continence benefit, not definitive long-term superiority.
**Study-level evidence from original clinical studies in men undergoing or following radical prostatectomy**						
Singh et al. [15]	Randomized trial; 41 randomized, 38 analysed; direct prostatectomy evidence, but intervention was general exercise rather than PFMT-specific.	Men with prostate cancer undergoing prostatectomy; predominantly robot-assisted laparoscopic prostatectomy. Baseline continence status was not the primary entry criterion; UI assessed postoperatively.	Six-week supervised prehabilitation before surgery: resistance, aerobic and trunk/core exercise three times weekly, approx. 90 min sessions. Attendance: 73.7% prehabilitation and 93.7% rehabilitation.	Identical exercise programme initiated postoperatively as rehabilitation 6 weeks after surgery.	UI assessed using 24 h pad test at 2, 6 and 12 weeks post-surgery. No significant between-group difference in urinary incontinence. Main benefits related to strength and functional outcomes rather than continence.	No exercise-related adverse events reported. Limitations: small sample, short follow-up, intervention not targeted PFMT, not powered primarily for continence. Interpretation: useful for broader physical-function recovery, not for PFMT efficacy.
Allameh et al. [16]	Randomized controlled trial; N = 57, with three groups of 19 participants; direct evidence concerning the timing of pelvic floor muscle biofeedback after radical prostatectomy.	Men undergoing radical prostatectomy. Baseline continence status was not clearly reported.	Group 1: pelvic-floor EMG biofeedback before surgery, 30 min twice weekly for 2 weeks. Group 2: no preoperative BF; pelvic-floor biofeedback after Foleycatheter removal, 30 min twice weekly for 2 weeks. Group 3: control/no biofeedback; non-functional probes before and after surgery. All groups received PFMT and 24 h pad-use instructions after catheter removal.	Control group (n = 19): nonfunctional probes were used before and after surgery. After Foley catheter removal, all patients received standard PFMT and 24 h pad-use instructions.	Outcome: pads/day at 1, 3 and 6 months after catheter removal. Continence at 1 month after catheter removal: 63% (group 1), 52% (group 2), and 15% (control); *p* = 0.012). At 3 months: 94% (group 1), 89% (group 2), and 52% (control), respectively (*p* = 0.01). At 6 months: 94% (group 1), 94% (group 2), and 83% (control), respectively (*p* = 0.20). Mean time to continence was 35.0 ± 8.7, 31.3 ± 6.5, and 50.7 ± 6.1 days, respectively; the control group recovered significantly later (*p* <0.001), whereas the difference between the two biofeedback groups was not significant (*p* = 0.27).	Adverse events: lost one patient to COVID-19 in biofeedback prior to the surgery group; lost contact with one patient in the no-intervention group. Limitations: small single-centre trial, limited protocol/adherence detail, possible unclear blinding. Interpretation: suggests biofeedback may accelerate early recovery, but optimal timing and long-term advantage remain uncertain.
Oh et al. [17]	Randomized controlled trial; N = 84, 42 per group; direct personalized extracorporeal biofeedback-assisted PFMT evidence.	Men after robot-assisted radical prostatectomy. Early postoperative PPI after RARP; incontinence severity was measured by the 24 h pad test.	Personalized extracorporeal biofeedback device for PFMT plus verbal and written instruction.	Verbal and written PFMT instruction only.	Outcomes at 1, 2 and 3 months: 24 h pad test, IPSS and IIEF-5. At 1 month, urine loss was lower with biofeedback-assisted PFMT (71.0 g vs 120.8 g; *p* = 0.028); no significant between-group difference from 2 months onward. IPSS favoured intervention at 1 month; IIEF-5 did not differ.	Adverse events: NR. Limitations: benefit early and not sustained, device-specific intervention, unclear long-term superiority. Interpretation: early adjunctive benefit only.
Laurienzo et al. [18]	Randomized controlled trial; N = 123; control n = 40, home PFMT n = 41, PFMT + electrical stimulation n = 42; direct PFMT/electrical stimulation evidence.	Men with prostate cancer treated by radical prostatectomy; randomized 1 month after RP. Assessments were performed preoperatively and postoperatively.	PFMT home-exercise guideline or PFMT plus anal electrical stimulation twice weekly for 7 weeks (35 Hz frequency, 1 ms pulse width, 2 s rise time, 6 s stimulation, 2 s fall time and 12 s rest; intensity was adjusted to produce visible pelvic floor contraction without discomfort). Adherence assessment: NR.	Control group without active PFMT/electrical-stimulation protocol.	Primary: pelvic floor strength. Secondary: 1h pad test, ICIQ-SF, IIEF-5 and IPSS at 1, 3 and 6 months. Leakage and pelvic floor weakness improved over time in all groups, but there was no significant between-group difference; PFMT or ES did not affect urinary continence recovery or erectile function in this trial.	Adverse events: NR. Limitations: spontaneous recovery may confound interpretation, no clear additive effect of ES, subgroup effects and adherence require verification. Interpretation: does not support routine ES add-on benefit in this context.
Goode et al. [19]	Multicentre randomized controlled trial; N = 208; direct evidence for behavioural therapy/PFMT in persistent postprostatectomy incontinence.	Community-dwelling men aged 51–84 years with urinary incontinence persisting 1–17 years after radical prostatectomy. This represents persistent/chronic UI, not early postoperative recovery.	Eight weeks of behavioural therapy delivered in 4 visits 2 weeks apart, including PFMT, bladder-control strategies, fluid management, daily home exercises, diaries and logs. Active treatment adherence was high and benefit was followed to 12 months.	Delayed-treatment control; behavioural therapy alone vs. behavioural therapy plus biofeedback and pelvic floor electrical stimulation.	Primary: reduction in weekly incontinence episodes from 7-day bladder diary at 8 weeks; durability to 12 months. Episodes/week decreased by 55% with behavioural therapy, 51% with behavioural therapy + biofeedback/electrical stimulation, and 24% in delayed-treatment controls. Active therapy was superior to control, but additional biofeedback/ES was not superior to behavioural therapy alone.	Two transient cases of hemorrhoidal irritation in the ES group. Limitations: chronic persistent PPI population, behavioural package rather than isolated PFMT, no added benefit from devices. Interpretation: supports behavioural/PFMT-based therapy for persistent PPI.
Li et al. [20]	Retrospective multicentre cohort study with propensity-score overlap weighting; adjusted analysis included 389 men; direct neuromodulation evidence but non-randomized.	Men with post-radical prostatectomy incontinence treated in three centres. Preoperative incontinence was excluded; baseline included at least 2 UI episodes/week in a 7-day diary. Prostatectomy type: NR.	Protocol- electrical EPNS: 60 min sessions with electrical stimulation via four sacrococcygeal needles; home PFMT was suspended. Duration: 3 sessions/week for 8 weeks. Adherence: missed sessions were rescheduled; actual adherence rates were not reported.	PFMT combined with transrectal electrical stimulation: PFMT + TES: 20 min EMG biofeedback-assisted PFMT + 20 min TES, plus home PFMT (30 contractions, 3 times/day).	Outcomes: ICIQ-UI SF, 24 h pad-test urine loss, IIQ-7 and pad-free status at 6 months after treatment. Adjusted analyses favoured EPNS: ICIQ-UI SF beta = −4.34; 24 h pad-test beta = −631.26 g; IIQ-7 beta = −2.86. Pad-free at last follow-up: 32.6% vs. 7.0%.	No serious treatment-related complications reported in accessible source; transient perineal soreness reported. Limitations: non-randomized retrospective design, possible residual confounding, treatment selection bias. Interpretation: promising, but prospective trials are needed before routine recommendation.
Niu et al. [21]	Prospective single-centre randomized, single-blind, sham-controlled clinical trial; N = 110 randomized, 101 completed follow-up; direct electroacupuncture evidence.	Men with localized prostate cancer after robot-assisted RP and early UI. UI defined as use of ≥2 pads/day at 4–6 weeks postoperatively; history of UI before RP was excluded.	Electroacupuncture at bilateral Ciliao, Zhongliao and Xialiao acupoints with 2/15 Hz stimulation, 30 min/session, 3 times/week for 6 weeks. Adherence reflected by treatment completion.	Sham electroacupuncture using nonpuncturing flat-tip needles at offset points.	Primary outcome: urinary continence recovery at week 6, defined as 0–1 pad/day. Continence: 43.6% (24/55) with electroacupuncture vs. 21.8% (12/55) with sham; RR = 2.00, 95% CI 1.12–3.59; *p* = 0.02. The 24 h urine leakage reduction also favoured electroacupuncture. Follow-up extended to 20 weeks.	Adverse events occurred in 4.0% of participants in the electroacupuncture group and 1.8% in the sham group; reported events included needle-insertion pain and subcutaneous hemorrhage. Limitations: single centre, selected early post-RARP population, follow-up still relatively short. Interpretation: promising adjunctive evidence requiring replication, not established standard care.
**(B). Indirect Radiofrequency/TECAR-Related Evidence from Non-Post-Prostatectomy Populations**						
**Study/Manuscript Reference**	**Design and Population**	**Intervention and Comparator**	**Outcomes and Main Findings**	**Why This Is Indirect Evidence for Post-RP Incontinence**		**Recommended Interpretation in the Revised Manuscript**
Elhosary et al. [22]	Pilot randomized controlled trial in 40 women with stress urinary incontinence; allocated 20/20. No post-prostatectomy population.	Monopolar capacitive-resistive radiofrequency (448 kHz) plus pelvic floor exercises vs. placebo radiofrequency application plus pelvic floor exercises.	Outcomes included pelvic-floor strength and symptom measures. Both groups improved, with greater improvement in the active radiofrequency plus exercise group. The continence outcome definition was not clearly reported. Adverse events were not systematically reported.	Female SUI population, different anatomy/pathophysiology and no men after radical prostatectomy; evidence is indirect and cannot establish efficacy for postprostatectomy UI.		Use only as hypothesis-generating/mechanistic support for possible RF/TECAR effects in pelvic-floor dysfunction. Do not state or imply clinical efficacy after radical prostatectomy.
Franic et al. [23]	Single-arm pilot study; N = 19 patients with overactive bladder and urgency urinary incontinence. No post-prostatectomy subgroup.	Radiofrequency therapy, four weekly 20 min sessions; no control/sham comparator.	ICIQ-OAB scores and the distribution of symptom severity improved after four treatment sessions, with the greatest improvement observed for urgency-related symptoms.	OAB/UUI population rather than male post-RP stress urinary incontinence; small uncontrolled sample and very short follow-up.		Mention only as preliminary indirect feasibility/physiological evidence. It should be clearly separated from direct post-RP continence rehabilitation evidence.

Notes for (A). AE = adverse event; BF = biofeedback; ES = electrical stimulation; EPNS = electrical pudendal nerve stimulation; ICIQ-UI = International Consultation on Incontinence Questionnaire-Urinary Incontinence; IIQ-7 = Incontinence Impact Questionnaire-7; IPSS = International Prostate Symptom Score; NR = not reported or not clearly reported in the accessible source used for table preparation; PFMT = pelvic floor muscle training; PPI = post-prostatectomy incontinence; RP = radical prostatectomy; RARP = robot-assisted radical prostatectomy; TES = transrectal/transanal electrical stimulation; UC = urinary continence.

**Table 2:** Characteristics of physiotherapy interventions used following radical prostatectomy.

Intervention	Target Population	Evidence Type	Timing/Objective	Interpretation	Safety/Barriers	Limitations
PFMT [13,29,32,33,40,43]	Men after RP.	Systematic reviews, retrospectivecomparative study.	Improve strength, endurance, coordination and rapid pelvic floor activation.	PFMT remains a reasonable core component of conservative rehabilitation and may accelerate early continence recovery; however, the magnitude and durability of benefit vary across protocols and populations.	Adverse events were not consistently reported; implementation depends on correct instruction and adherence.	Protocol heterogeneity; variable adherence; inconsistent outcome definitions.
Preoperative/perioperative pelvic-floor rehabilitation and exercise [14,15,45]	Men undergoing RP.	Clinical trials and reviews.	Facilitate earlier continence recovery; potentially improve postoperative outcomes.	Preoperative PFMT may facilitate earlier continence recovery in selected patients, but evidence is heterogeneous. General prehabilitation exercise should not be interpreted as evidence specific to PFMT.	Adverse events were not consistently reported; practical barriers include adherence and the need to complete training before surgery.	Heterogeneous protocols; uncertain long-term benefit; variable timing.
PFMT + EMG biofeedback [16,17]	Men after RP.	RCTs.	Facilitate correct muscle activation and motor learning.	May be a useful adjunct; particularly when patients have difficulty identifying or correctly activating the pelvic floor muscles; should not be considered a replacement for PFMT.	Adverse events were not clearly reported; implementation may be limited by equipment availability, professional expertise and adherence.	Small/heterogeneous studies; technical variation.
Electrical stimulation [18,19,46,47]	Men after RP.	RCTs and systematic reviews.	Facilitate pelvic floor activation and potentially reduce leakage.	Current evidence does not demonstrate a consistent additive benefit of electrical stimulation over PFMT or behavioural therapy alone. Results remain heterogeneous and protocol-dependent.	Adverse events were inconsistently reported, and the available studies do not allow a reliable assessment of treatment safety; barriers include equipment requirements.	Variable parameters, electrode placement and treatment duration; study-quality limitations.
Nerve stimulation [20]	Men after RP.	Retrospective cohort study; limited evidence in the reviewed literature.	Neuromodulation aimed at improving urinary continence in men with persistent post-prostatectomy incontinence.	Preliminary short-term findings suggest potential benefit, but evidence from a single retrospective cohort is insufficient to support routine clinical use.	Reported adverse events included transient perineal soreness; no serious treatment-related complications were reported.	Limited direct evidence in post-prostatectomy SUI; specialized/invasive intervention; limited follow-up; long-term effectiveness uncertain.
Electroacupuncture [21]	Men after RP.	RCT; limited evidence in the reviewed literature.	Reduce early postoperative urinary incontinence.	Promising adjunctive intervention; preliminary direct evidence suggests potential benefit, but replication is required before routine use.	Adverse events occurred in 4.0% of participants in the electroacupuncture group and 1.8% in the sham group; reported events included needle-insertion pain and subcutaneous hemorrhage. Requires trained personnel and repeated treatment sessions (3×/week for 6 weeks).	Evidence currently based on a single directly relevant RCT; single-centre population and relatively limited follow-up; replication required.
TECAR/ radiofrequency [22,23]	Evidence largely from non-post-RP populations.	Indirect evidence.	Proposed tissue and neuromuscular effects.	Experimental and hypothesis-generating; no direct clinical evidence supports efficacy for urinary incontinence in men after RP.	Safety for men after RP cannot be established from the available indirect evidence. Adverse-event reporting in the cited RF studies was insufficient to support a reliable safety conclusion for the target population.	Wrong/indirect populations; limited clinical evidence; parameters and safety require clarification.

Abbreviations: PFMT—pelvic floor muscle training; RP—radical prostatectomy; EMG—electromyography; RCT—randomized controlled trial; SUI—stress urinary incontinence; TECAR—transfer energy capacitive and resistive.

## Data Availability

No new data were created or analyzed in this study. Data sharing is not applicable to this article.

## Abbreviations

SUI	Stress urinary incontinence
BMI	Body mass index
PFMT	Pelvic floor muscle training
EMG	Electromyography
FES	Functional electrical stimulation
